# Identification of Differentially Expressed Genes in Different Types of Broiler Skeletal Muscle Fibers Using the RNA-seq Technique

**DOI:** 10.1155/2020/9478949

**Published:** 2020-07-04

**Authors:** Han Wang, Zhonghao Shen, Xiaolong Zhou, Songbai Yang, Feifei Yan, Ke He, Ayong Zhao

**Affiliations:** College of Animal Science and Technology, ·College of Veterinary Medicine, Zhejiang A&F University, Lin'an, Zhejiang 311300, China

## Abstract

The difference in muscle fiber types is very important to the muscle development and meat quality of broilers. At present, the molecular regulation mechanisms of skeletal muscle fiber-type transformation in broilers are still unclear. In this study, differentially expressed genes between breast and leg muscles in broilers were analyzed using RNA-seq. A total of 767 DEGs were identified. Compared with leg muscle, there were 429 upregulated genes and 338 downregulated genes in breast muscle. Gene Ontology (GO) enrichment indicated that these DEGs were mainly involved in cellular processes, single organism processes, cells, and cellular components, as well as binding and catalytic activity. KEGG analysis shows that a total of 230 DEGs were mapped to 126 KEGG pathways and significantly enriched in the four pathways of glycolysis/gluconeogenesis, starch and sucrose metabolism, insulin signalling pathways, and the biosynthesis of amino acids. Quantitative real-time reverse transcription polymerase chain reaction (qRT-PCR) was used to verify the differential expression of 7 selected DEGs, and the results were consistent with RNA-seq data. In addition, the expression profile of *MyHC* isoforms in chicken skeletal muscle cells showed that with the extension of differentiation time, the expression of fast fiber subunits (types IIA and IIB) gradually increased, while slow muscle fiber subunits (type I) showed a downward trend after 4 days of differentiation. The differential genes screened in this study will provide some new ideas for further understanding the molecular mechanism of skeletal muscle fiber transformation in broilers.

## 1. Introduction

Chicken is widely welcomed by consumers because it is low in fat, is low in cholesterol, and has no religious restrictions. Therefore, chicken has become the second-largest consumed meat product after pork [1, 2]. In recent years, with the continuous improvement in people's living standards, their requirements for meat flavor have also become stricter. Age, sex, heredity, environment, nutrition, and intramuscular fat content (IMF) are all important factors affecting chicken quality [3–6]. In addition, studies have shown that muscle fiber properties were an essential factor affecting the meat quality of chicken [7, 8]. Skeletal muscle is one of the most important components of meat-producing animals, accounting for approximately 45%~60% of the whole animal body [9]. According to the characteristics of contraction and metabolism, skeletal muscle fibers can be roughly divided into two types: slow-twitch (type I) and fast-twitch (type II) muscles. Type II muscle fibers can be categorized into a fast-twitch oxidation type (IIA), a fast-twitch glycolytic type (IIB), and a super-fast-twitch type (IIX) [10, 11]. Previous studies showed that muscle with a high proportion of fast-twitch muscle fibers appeared paler, while the muscle with more slow-twitch muscle fibers tended to be redder [8]. Therefore, skeletal muscle was usually divided into two kinds of muscle, red muscle and white muscle [12]. Moreover, it was found that muscles with a higher proportion of oxidized muscle fibers had better meat quality [13].

Currently, it has become a research hotspot to study the molecular mechanism of muscle fiber-type transformation in order to improve meat quality. It has been shown that *PGC-1* can promote the transformation of muscle into type I fibers in the skeletal muscle [14]. Cofilin2b (*CFL2b*) can affect the transformation pattern of myosin heavy chain (*MyHC*) subunits in the muscle of piglets [15]. Furthermore, noncoding RNA was also proven to play a role in slow-twitch muscle fiber formation and skeletal myogenesis [16]. At present, preliminary progress has been made on the molecular mechanism of chicken muscle fiber-type transformation. One study showed that the expression of *PGC-1α* might be closely related to the slow muscle fiber content in chicken skeletal muscle [17]. In addition, miR-1611 was discovered to be highly expressed in slow-twitch muscle fibers and could drive the transformation from fast-twitch to slow-twitch muscle fibers in chicken [18]. However, the regulatory network of muscle development is complex, and studies on the formation and transformation of chicken skeletal muscle fiber types are still incomplete. Therefore, more relevant, important genes need to be further explored.

At present, transcriptome sequencing technology has been widely used in biological research, medical diagnostics, and therapeutic studies [19]. Meanwhile, this technique also plays an important role in farm animal muscle development. Based on the transcriptome analysis of biceps between Small-tail Han sheep and Duper sheep, a total of 1300 differentially expressed genes were identified, which could help further elucidate the mechanisms of muscle development as they are affected by breed [20]. Forty-nine differentially expressed genes were screened from the longissimus dorsi of Nellore cattle among different grades of marbling by RNA-seq, in an effort to find strategies to select animals with greater marbling [21]. Moreover, another study used RNA-seq to identify genes related to pig muscle development and meat quality [22]. RNA-seq technology was also used in broiler chicken groups to uncover 68 genes that might participate in the tenderization process [23]. Thus, it can be seen that whole-genome sequencing technology exerts an immense influence on the discovery of new functional genes.

To better explore the molecular mechanism of skeletal muscle fiber transformation in broilers, this study used RNA-seq technology to screen differentially expressed genes between breast and leg muscles of Ross 308 broiler chickens. Subsequently, through GO and KEGG pathway analysis, genes and pathways related to skeletal muscle fiber-type conversion were discovered. In addition, to further explore the growth characteristics of broiler muscle fibers, a skeletal muscle cell differentiation model of broilers was constructed in vitro. Subsequently, the expression of *MyHC* subunits during the differentiation of chicken skeletal muscle satellite cells was studied. These results will provide a theoretical basis for the genetic improvement in muscle quality in broilers in the future.

## 2. Materials and Methods

### 2.1. Experimental Animals and Tissues

The chickens used in this study were Ross 308 broiler chickens from a farm in Fuyang District, Hangzhou, Zhejiang Province, China. Five 42-day-old Ross 308 broilers with similar body weights were selected for slaughter. The leg and breast muscles were collected and frozen at -80°C for RNA extraction.

At the same time, we bought 15 fertilized eggs of the same breed and incubated them for 10 days to collect chicken skeletal muscle satellite cells. All animal procedures used in this study were approved by the Ethics Committee for Animal Experiments of Zhejiang A&F University and were performed in accordance with the Guidelines for Animal Experimentation of University (Hangzhou, China).

### 2.2. Total RNA Extraction, Library Construction, and Sequencing

Total RNA was extracted from the gastrocnemius of legs and the pectoralis major of breasts using the TRIzol method [24]. The quality inspection was carried out through the Sample Testing Center of Biomarker Technologies Corporation (Beijing, China) to ensure that the samples were up to the standard, and the test quality report of the RNA samples is presented in Table S1. All RNA samples with an OD 260/280 1.8-2.0 qualified for further analysis. Sequencing libraries were generated using a NEBNext Ultra™ RNA Library Prep Kit (NEB, USA). Briefly, mRNA was purified from 1 *μ*g of total RNA per sample using poly-T oligoattached magnetic beads. Then, first-strand cDNA was synthesized using a random hexamer primer and M-MuLV Reverse Transcriptase. Second-strand cDNA synthesis was subsequently performed using DNA polymerase I and RNase H. The cDNA fragments of 240 bp were preferentially selected with an AMPure XP system (Beckman Coulter, USA) for cDNA adaptor ligating. At last, polymerase chain reaction (PCR) amplification was carried out and the products were purified (AMPure XP system). The library quality was assessed on the Agilent Bioanalyzer 2100 system. The libraries were sequenced by paired-end (PE 150 bp) sequencing on an Illumina HiSeq™ platform (Illumina, USA), and the raw reads were obtained. The leg muscle and breast muscle tissues of broilers were numbered PT1-PT5 and PX1-PX5, respectively, for subsequent analysis.

### 2.3. Sequencing Quality Assessment and DEG Screening

Clean data (clean reads) was obtained by removing reads containing adapters or poly-N; low-quality reads were also removed from the raw data. At the same time, Q20, Q30, GC-content, and sequence duplication levels of the clean data were calculated. Ultimately, all downstream analyses were based on high-quality clean data. The chicken genome sequence (GRCg6a) was downloaded from the NCBI database (https://www.ncbi.nlm.nih.gov/genome/?term=chicken). Then, Hisat2 software was used to map these clean reads to the chicken reference genome [25]. Transcript assembly was performed using StringTie software [26]. StringTie is an algorithm based on optimization theory, which uses comparison information to construct a multivariable clipping graph and traffic network to assemble and evaluate the expression of reads according to the maximum flow algorithm. Compared with other software such as Cufflinks, it can build a more complete transcript and better evaluate expression [26]. Quantification of gene expression levels was estimated by the fragments per kilobase of transcript per million fragments (FPKM) method [27]. Based on raw counts, the reads mapped to the reference genome were quantified by StringTie software to obtain FPKM [28]. Then, DESeq R package (1.39.0), a statistical program for determining differential expression in digital gene expression data based on a model of negative binomial distribution, was used to analyze the differential gene expression between samples with biological repetition [29]. Differentially expressed genes (DEGs) were identified using the following filter criteria: fold change ≥ 2 and FDR < 0.05. Fold change indicates the ratio of expression quantity between two samples (groups). The false discovery rate (FDR) was obtained by correcting the significant difference *P* value.

### 2.4. Gene Ontology (GO) and KEGG Pathway Enrichment Analysis

Gene Ontology (GO) enrichment analysis of the DEGs was implemented by GOseq R packages (http://www.geneontology.org/) based on Wallenius noncentral hypergeometric distribution [30], which could adjust for gene length bias in DEGs. Pathway significant enrichment analysis was run based on the hypergeometric test in the KEGG database (http://www.genome.jp/kegg/) to uncover the most significantly enriched pathways among the DEGs. KOBAS software [31] was used to test the statistical enrichment of DEGs in KEGG pathways.

### 2.5. Quantitative Real-Time RT-PCR Verification

Utilizing the NCBI gene bank, primers of 7 randomly selected DEGs, three different isoforms of *MyHC* genes (*MyHC* I, IIA, and IIB) and the house-keeping gene *GAPDH* were designed by Primer Premier 5 software (Table S1). Primers were synthesized by Hangzhou Youkang Biotechnology Co., Ltd. (Zhejiang, China). Total RNA from cells or tissues was used to synthesize cDNA by a 5X All-In-One MasterMix Kit (abm, China). The cDNA was used as a template for quantitative PCR by using the EvaGreen 2X qPCR MasterMix Kit (abm, China). There were three replicates for each sample. The relative expression level of each related gene was calculated using the 2^-*△△*CT^ statistical analysis method.

### 2.6. Isolation and Culture of Chicken Skeletal Muscle Satellite Cells In Vitro

The 10-day fertilized eggs were disinfected with ethanol. The eggshell on the upper part of the air chamber was removed using tweezers on the egg receptacle. A new pair of tweezers was used to clip out the chicken embryo. The leg muscle of the chicken embryo was isolated and placed in a new petri dish. Muscle samples were washed 3 times in phosphate buffer solution (PBS) (Logan, USA) containing penicillin-streptomycin liquid (Solarbio, China), and skin, blood vessels, adipose tissue, and connective tissue were removed. The sterile muscle tissue was cut into meat paste and digested with 0.25% trypsin-EDTA (Thermo Fisher, China) at 37°C for 20 min. DMEM/F12 (Logan, USA) containing 10% fetal bovine serum (FBS, Thermo Fisher, China) was added to terminate the digestion. The suspension was filtered through a 70 *μ*m mesh sieve and centrifuged at 1200 r/min for 8 min at room temperature. As the supernatant was discarded, cells were resuspended with DMEM/F12 containing 15% FBS and cultured in a 5% CO_2_ incubator at 37°C. One hour later, the cell suspension was transferred into a new cell petri dish; this was repeated twice to enrich for muscle satellite cells and eliminate fibroblasts. Cell differentiation was induced by replacing 15% FBS with 2% equine serum (Logan, USA). Once the isolated skeletal muscle satellite cells reached 70%-80% fusion, the growth medium was replaced with differentiation medium and the cells were cultured for 2, 4, and 6 days. Cells at 70%-80% fusion indicated day 0.

### 2.7. Immunofluorescence Staining

The isolated skeletal muscle satellite cells at 70%-80% fusion and the cells that were induced to differentiate after transfection were used for immunofluorescence detection following the method of Luo et al. [32]. Briefly, cells grown in 12-well plates were washed three times with precooled PBS and fixed with 4% paraformaldehyde for 15 min. Thereafter, the cells were permeabilized with 0.25% Triton X-100 per well for 10 min and blocked at 4°C overnight. Afterwards, cells were incubated with 1 : 100 diluted primary anti-*Desmin* (Abcam, China), anti-*Pax7* (Abcam, China), or anti-*MyHC* (Santa, USA) antibodies for 1 hour at room temperature. Then, 1 : 100 diluted fluorescent secondary antibody (Thermo Fisher, China) was incubated with the cells for 1 hour at room temperature. After being washed by PBS three times, DAPI (Invitrogen, USA) was added to the cells and then incubated for 15 min at room temperature to stain the cell nuclei. Lastly, samples were captured using a fluorescence microscope (OLYMPUS, Japan).

## 3. Results

### 3.1. Expression of MyHC Genes in Different Muscle Tissues of Broilers

**Figure 1:**
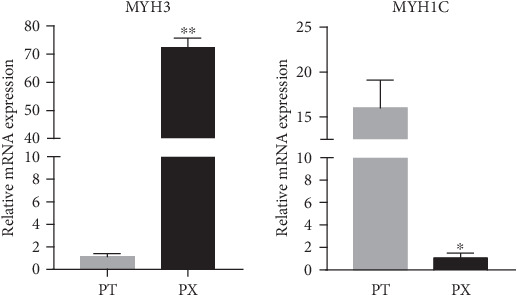
Expression of *MyHC* genes in different muscle tissues of broilers. Fast muscle marker gene *MYH3* and slow muscle marker gene *MYH1C*. ^∗∗^An extreme significant difference between the two groups (*P* < 0.01); ^∗^a significant difference between the two groups (*P* < 0.05); PT represents leg muscle; PX represents breast muscle, *n* = 5.

In some previous studies, compared with histochemical methods, qRT-PCR was a fast and sensitive approach to distinguish different muscle types by identifying the expression of *MyHC* subunits [33, 34]. This method was also applied to identify the muscle types of chickens [35, 36]. Therefore, in order to compare the fiber types of different muscles in broilers, the expression of *MYH3*, a fast muscle fiber marker gene [37], and *MYH1C*, a slow muscle fiber marker gene [38], were detected by qRT-PCR between breast and leg muscles of 10 randomly selected broilers. The results showed that the expression of *MYH3* in the breast muscle was significantly higher than that in the leg muscle, while the expression of *MYH1C* showed the opposite result (Figure 1). The results indicated that the breast and leg muscles of broilers were two different types of skeletal muscle. The former was mainly composed of slow muscle fibers, while the latter was mainly composed of fast muscle fibers.

### 3.2. Sequencing Quality Evaluation

To verify the authenticity of the sequencing data, samples were tested for quality. In Table 1, it could be found that the Q20 (a base quality > 20 and error rate < 0.01) and Q30 (a base quality > 30 and error rate < 0.001) levels were both above 90%. Meanwhile, the GC content was between 49% and 53%. The results showed that the sequencing data had a small probability of error.

It was also found that more than 75% of reads aligned to the reference genome and that the number of reads aligned to a unique location of the reference genome was between 74% and 92%, which indicated that these samples were highly available (Table 2). To summarize, the sequencing data were good and the subsequent analysis could be carried out.

### 3.3. Screening of DEGs

**Figure 2:**
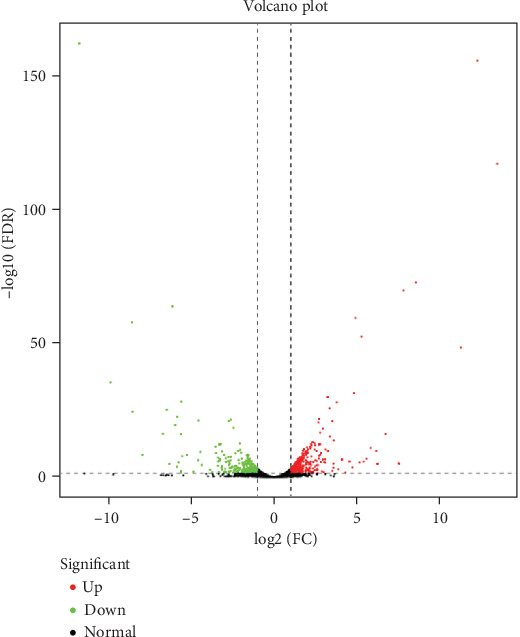
Volcano plot for differential gene expression.

To show the distribution of gene expression, a volcano plot of the differentially expressed genes was constructed, with -log10(FDR) as the ordinate and log2(FC) as the abscissa. Because the differential expression analysis of transcriptome sequencing was an independent statistical hypothesis test for a large number of gene expression values, there was a false-positive problem. Therefore, in the process of differential expression analysis, the recognized Benjamini-Hochberg correction method was used to correct the significant *P* values obtained by the original hypothesis test. Finally, FDR was used as the key index for differentially expressed gene screening. In our study, FDR < 0.05 was used as a screening condition for DEGs. The results showed that there were a large number of differentially expressed genes. A total of 767 DEGs were identified. Compared with leg muscle, there were 429 upregulated genes and 338 downregulated genes in the breast muscle (Figure 2 and Table S2).

**Figure 3:**
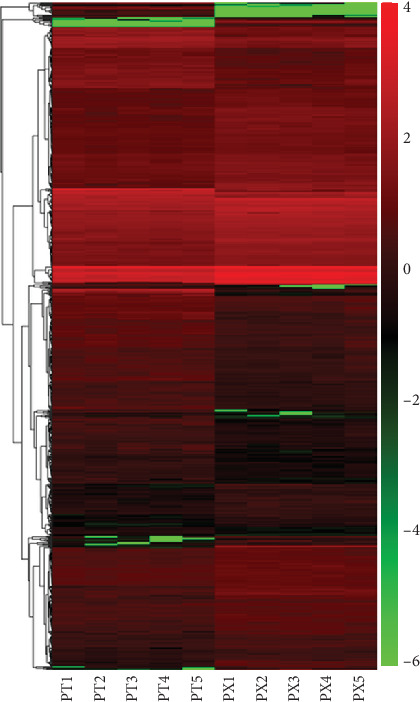
The heat map of differential gene expression (PT represents broiler leg muscle; PX represents breast muscle).

Meanwhile, in order to ensure the accuracy of the analysis, the screened differentially expressed genes were analyzed by hierarchical cluster analysis where genes with the same or similar expression patterns clustered. The abscissa represented the sample clustering. Among them, one column represents a sample, and clustering is based on the similarity of gene expression between samples. The more similar the gene expression between samples, the closer the distance. This result suggested that these DEGs could significantly separate the samples into the leg muscle group and breast muscle group (Figure 3), indicating the reliability of our analysis.

### 3.4. Gene Ontology (GO) Annotation and Enrichment Analysis

**Figure 4:**
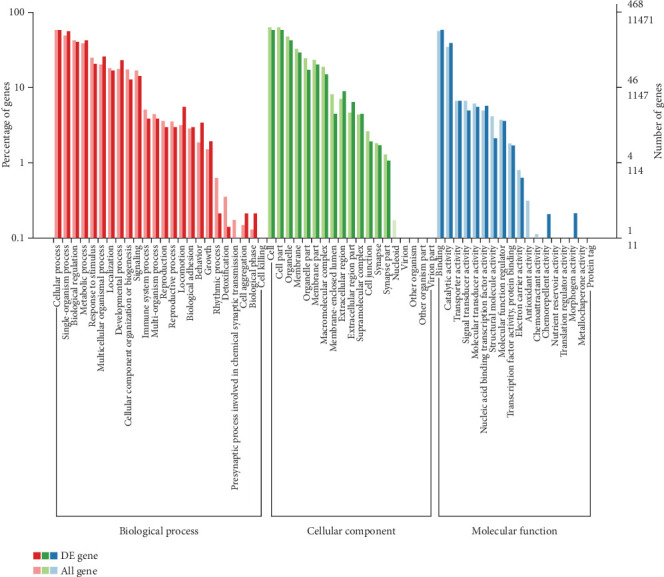
GO analysis of the DEGs.

To further reveal the molecular characterization of the DEGs, GO annotation was used to analyze the DEGs. The GO database contains three main branches: biological process, molecular function, and cellular component. The results showed that a total of 468 DEGs were annotated into 61 GO terms, including 24 biological processes, 19 cellular components, and 18 molecular function annotations. In biological processes, the significantly enriched GO terms mainly contained cellular process, single organism process, biological regulation, metabolic process, response to stimulus, multicellular organismal process, localization, developmental process, cellular component organization, biogenesis signalling, etc. Cells, cellular parts, and organelles were significantly enriched in cellular components. Simultaneously, the main functional terms of molecular function were binding and catalytic activity, which were enriched with 273 and 183 genes, respectively (Figure 4).

In addition, some important genes (FDR < 0.01) that may be involved in the transformation of muscle fiber types are enriched in these GO terms, such as mitotic cell cycle process (*FHL1*, *PDS5A*) and calcium ion binding (*NKD1*, *RYR3*) (Table S3).

### 3.5. KEGG Pathway Enrichment Analysis

**Figure 5:**
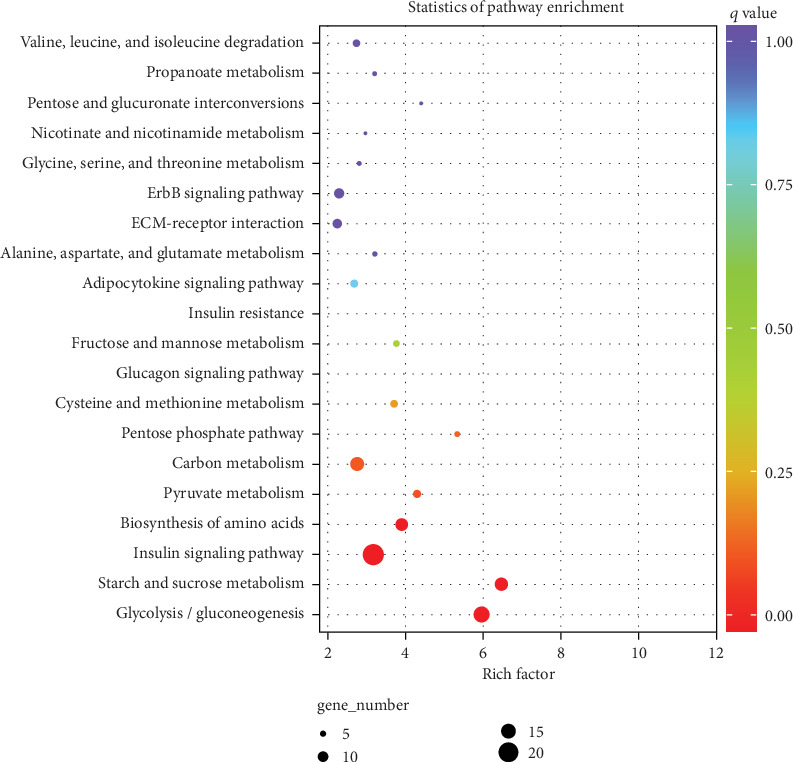
Top 20 significantly enriched KEGG pathways.

To know the signal pathways where the genes were distributed, a KEGG pathway analysis was performed. A total of 230 DEGs were mapped to 126 KEGG pathways, of which 20 pathways were significantly enriched (*P* ≤ 0.05) (Table S4). There were several extremely significantly enriched pathways (Figure 5), including glycolysis/gluconeogenesis, starch and sucrose metabolism, insulin signalling, and biosynthesis of amino acids. Some important genes were found to be enriched in these pathways: *PKM*, *LDHA*, *LDHA*, *PYGM*, and *HRAS* (FDR < 0.01) (Table S4).

### 3.6. Validation of DEGs by qRT-PCR

**Figure 6:**
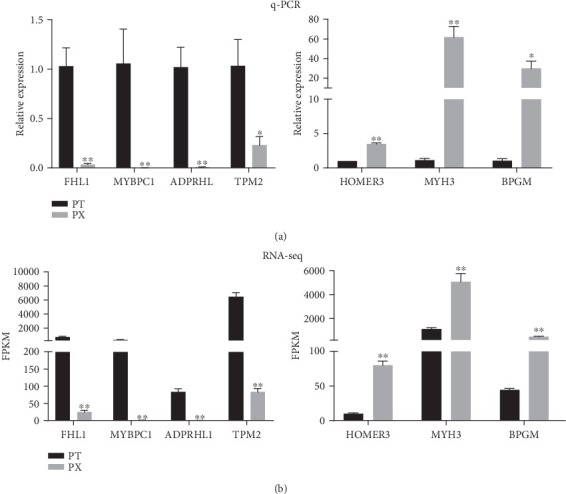
Validation of DEGs by qRT-PCR: (a) q-PCR results and (b) RNA-seq results (^∗∗^*P* < 0.01, ^∗^*P* < 0.05; PT represents broiler leg muscle; PX represents breast muscle, *n* = 5).

The expression of 7 randomly selected DEGs, including 4 downregulated genes (*FHL1*, *MYBPC1*, *ADPRHL*, and *TPM2*) and 3 upregulated genes (*HOMER3*, *MYH3*, and *BPGM*), was verified through quantitative real-time PCR. The results illustrated that the expression of *FHL1*, *MYBPC1*, *ADPRHL*, and *TPM2* in the breast muscle was significantly lower than that in the leg muscle, while the expression of *HOMER3*, *MYH3*, and *BPGM* was exactly the opposite (Figure 6(a)). As shown in Figure 6, the expression trend of these 7 genes was consistent with the results of the RNA-seq experiment, which proved that the RNA-seq results were reliable (Figure 6(b)).

### 3.7. Identification of Chicken Skeletal Muscle Satellite Cells

**Figure 7:**
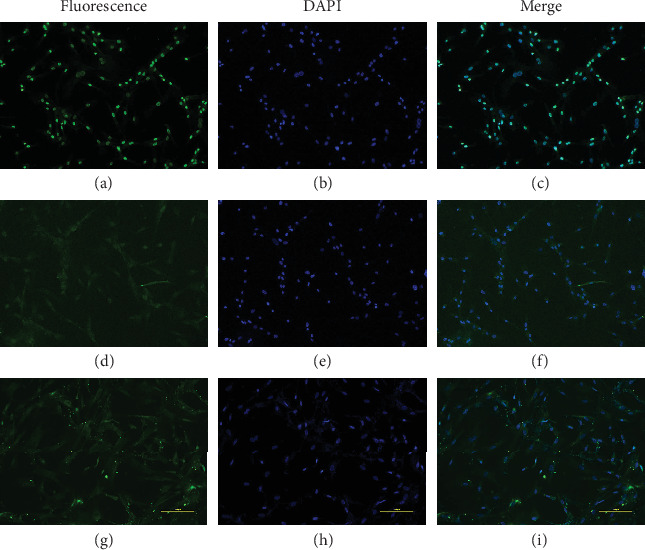
Identification of skeletal muscle satellite cells: (a) *Pax7* was detected with immunofluorescence; (b) DAPI stain of the nuclei of skeletal muscle satellite cells; (c) merged image of (a) and (b); (d) *Desmin* was detected with immunofluorescence; (e) DAPI stain of the nuclei of skeletal muscle satellite cells; (f) merged image of (d) and (e); (g) *MyHC* was detected with immunofluorescence; (h) DAPI stain of the nuclei of skeletal muscle satellite cells; (i) merged image of (g) and (h).

To facilitate the study of muscle fiber growth characteristics in broilers, we isolated skeletal muscle satellite cells from the leg muscle of 15 chicken embryos which were incubated for 10 days in vitro. The cells in the process of proliferation and differentiation were identified by immunofluorescence with the skeletal muscle satellite cell-specific markers *Pax7*, *Desmin*, and *MyHC* [39]. The results showed that *Pax7* was positive in the nucleus (Figure 7(a)), and *Desmin* and *MyHC* were positive in the cytoplasm (Figures 7(d) and 7(g)). Therefore, the results showed that the isolated cells are indeed skeletal muscle satellite cells and could be used in the following research.

### 3.8. Expression Profile of MyHC Genes

**Figure 8:**
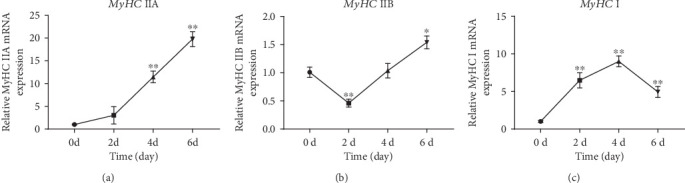
Expression of *MyHC* genes in the differentiation of chicken skeletal muscle satellite cells (^∗∗^*P* < 0.01, ^∗^*P* < 0.05; *n* = 3).

In order to further explore the growth of muscle fibers in broilers, the expression patterns of *MyHC* subunits during cell differentiation were analyzed by qRT-PCR. *MyHC* IIA is a genetic marker of fast oxidative muscle fibers [40]. *MyHC* IIB is a genetic marker of fast glycolytic muscle fibers [41]. *MyHC* I is a genetic marker of slow oxidative muscle fibers [42]. The results suggested that the expression of *MyHC* IIA was significantly upregulated during cell differentiation (Figure 8(a)). However, the expression of *MyHC* IIB was significantly downregulated on the second day of differentiation and then gradually upregulated from day 4 to day 6 (Figure 8(b)). Additionally, the expression of *MyHC* I was significantly upregulated at the beginning and decreased after the 4th day of differentiation (Figure 8(c)). The results indicated different development of muscle fiber types in broilers.

## 4. Discussion

The skeletal muscle of vertebrates is composed of muscle fibers with different morphological, contractile, and metabolic characteristics. Meanwhile, the properties of muscle fibers have an important effect on the meat quality of livestock [8]. In our study, we found that slow/fast muscle fiber marker genes were differentially expressed between the breast and leg muscles of broilers. The results were consistent with several previous studies demonstrating that the breast muscle of the chicken was a fast muscle, while the leg muscle was a slow muscle [43]. Therefore, RNA-seq was performed on these two skeletal muscles to screen candidate genes related to muscle fiber-type conversion in our research. A total of 767 DEGs were discovered between both investigated groups, of which 7 DEGs were randomly selected and verified by qRT-PCR analysis. Although the expression fold changes of the selected genes between the two methods were not the same, the expression trend was highly consistent, proving that the results obtained by chip analysis were credible.

The basal processes of muscle cell growth and muscle fiber formation in broilers include cellular processes, single biological processes, and biological regulation. GO functional analysis showed that 11 DEGs were enriched in GO term mitotic cell cycle processes (Table S3). Among these DEGs, the *FHL1* gene was found to be highly abundant in oxidative muscle fibers [44]. Overexpression of *FHL1* promoted the transformation of muscle fibers to the oxidative type, while knockdown of *FHL1* could cause muscle fiber hypertrophy [45]. Slow muscle fibers are usually small in diameter, whereas the fast muscle fibers are usually large in diameter [46]. In this study, the *PDS5A* gene was also screened as an upregulated DEG in the breast muscle. *PDS5A* was found to be highly expressed in the skeletal muscle but low in other tissues and to have an effect on the proliferation of skeletal muscle cells [47]. These genes might play a key role in regulating muscle cell proliferation and muscle fiber transformation. In addition, intracellular calcium signalling is also an important factor that influences muscle fiber types [48]. Our study showed that 15 DEGs were enriched in the GO term calcium ion binding (Table S3). Among these genes, *NKD1* was upregulated in leg muscles. Some studies have shown that Wnt/*β*-catenin signalling could transform slow muscle fibers into fast muscle fibers and that *NKD1* has a strong antagonistic effect on Wnt/*β*-catenin signalling [49, 50]. Another gene, *RYR3*, controls calcium ion release channels in muscle cells [51]. *RYR3* has a high calcium sensitivity and regulates rapid muscle contraction together with the *RYR1* gene [52]. Some studies have shown that calcium sensitivity is related to the generation of fast myofibril subtypes, which was consistent with the results of our study [53]. KEGG pathway analysis also showed that the expression of calcium signalling pathway-related genes was altered significantly (Table S4). The function of these aforementioned DEGs in skeletal muscle fiber transformation needs further investigation.

One of the methods to classify fast or slow muscle is by their energy metabolism [54]. GO functional annotation analysis showed that a total of 198 DEGs were enriched in metabolic process-related GO terms (Table S3). Previous studies showed that slow-twitch muscles have lower glycolytic enzyme activity, while fast-twitch muscles act mainly through the glycolytic pathway to meet the energy requirements for rapid muscle contraction [55, 56]. In our study, the KEGG pathway enrichment analysis results indicated that the four most significant enrichment pathways were related to the energy metabolism of muscle cells. Most of the DEGs involved in the glycolysis/gluconeogenesis pathway were upregulated in the breast muscle (15 of 17) (Table S4). Protein synthesis during skeletal muscle development is accompanied by energy metabolism, and the glycolysis/gluconeogenesis pathway plays a significant role [57]. *PKM* is a gene that catalyses the last step of glycolysis [58] and was upregulated in the breast muscle. The muscle type of pyruvate kinase (*PKM*) is one of the key mediators of the Warburg effect and tumor metabolism [59] and was closely related to the development of PSE meat [60]. Another gene, *LDHA*, the predominant *LDH* isoform in the skeletal muscle, is also an important enzyme involved in glycolysis [61]. The mutation at the C.423A>G site in exon 6 of porcine *LDHA* had a significant effect on the muscle fiber type II ratio [62]. In addition, 13 DEGs were enriched in the biosynthesis of amino acid pathway, of which 12 DEGs were upregulated in the breast muscle (Table S4). Another study found that genes related to glycolysis/gluconeogenesis and the biosynthesis of amino acid pathway were gradually upregulated during the growth and development of chicken breast muscle [63]. Therefore, we inferred that these DEGs were activated during fast-twitch muscle fiber formation.

Our KEGG results further discovered that 22 DEGs were enriched in the insulin signalling pathway (Table S4). Insulin plays a key role in a series of biological reactions, such as glucose uptake, lipid and protein synthesis, cell growth, and glycogen synthesis [64]. In skeletal muscle cells, the insulin signalling pathway regulates glycogen synthesis and decreases the rate of glycogen breakdown [65]. It has been shown that the content of glycogen in type I skeletal muscle fibers is significantly lower than that in type II muscle fibers [66]. We found that 5 DEGs in the insulin signalling pathway were also enriched in the GO term glycogen metabolic process (Table S3), including *GYS1* and *PYGM*; these were all upregulated in the breast muscle. The insulin signalling pathway stimulates the expression of *GYS1* mRNA and promotes the synthesis of glycogen in the skeletal muscle [67]. Skeletal muscle fiber type IIB predominantly uses glycogen and glucose as fuel, and the upregulation of *PYGM* accelerates glycogen phosphorylation [68]. In addition, 3 DEGs in the insulin signalling pathway were upregulated in the leg muscle, including *HRAS* (Table S4). The expression of *HRAS* was negatively correlated with metabolic traits related to the insulin signalling pathway [69]. Thus, *HRAS* may be a candidate gene involved in slow muscle fiber formation.

Skeletal muscle fibers are generally divided into four types according to the myosin heavy chain (*MyHC*) isoforms, namely, types I, IIA, IIB, and IIX [70]. However, there are very few type IIX muscle fibers in poultry skeletal muscle [71]. A previous study showed that *Pax7*, *Desmin*, and *MyHC* were specific genetic markers of skeletal muscle satellite cells [72]. To further explore the mechanism of skeletal muscle fiber transformation in broilers, an in vitro cell model was successfully constructed in our study. Then, the expression profiles of *MyHC* isoforms (type I, type IIA, and type IIB) during broiler skeletal muscle satellite cell differentiation were detected. *MyHC* IIA mRNA expression increased throughout the time course. Expression of the *MyHC* IIB isoform was low on day 2 but rose until day 6. *MyHC* I expression first grew dramatically from day 0 to day 4 and then declined until day 6. Interestingly, these results coincided with those in a C2C12 mouse myoblast cell differentiation model [73]. Therefore, future studies will use these approximate time points of skeletal muscle fiber transformation in vitro to explore the function of the key DEGs in broilers.

## 5. Conclusion

In the current research, RNA-seq was carried out to compare the leg and breast muscles of broilers. A total of 767 DEGs were identified. Compared with leg muscle, there were 429 upregulated genes and 338 downregulated genes in the breast muscle. The expression profile of *MyHC* isoforms during broiler skeletal muscle cell differentiation was detected. Therefore, our study provides useful information for understanding the molecular mechanisms of muscle fiber-type transition in broilers. However, the transformation of skeletal muscle fiber in vivo is a dynamic process and these transcriptome data are preliminary, so the function of the DEGs still requires further investigation.

## Figures and Tables

**Table 1:** Quality assessment of sequencing data.

Sample name	Clean reads	Clean bases	GC (%)	Q20 (%)	Q30 (%)
PT1	29135911	8705716622	51.03	97.65	93.63
PT2	32518744	9699863244	51.19	97.74	93.87
PT3	32658211	9752147744	51.27	97.73	93.85
PT4	37753032	11278877720	52.40	97.92	94.24
PT5	34146017	10159903756	52.05	97.86	94.13
PX1	25454197	7590698526	51.36	97.11	92.33
PX2	33709265	10026742412	51.19	97.61	93.31
PX3	29558343	8794379722	51.21	97.29	92.71
PX4	25035478	7478569054	51.89	97.64	93.46
PX5	29461523	8807133318	51.89	97.57	93.37

**Table 2:** Statistical results of the comparison with the reference genome.

Sample name	Total reads	Mapped reads	Mapped ratio (%)	Uniquely map	Uniquely mapped ratio (%)	Reads mapped to “+” strand	Reads mapped to “+” strand ratio (%)	Reads mapped to “-” strand	Reads mapped to “-” strand ratio (%)
PT1	58271822	51290093	88.02%	48720157	83.61%	25470179	43.71%	25437454	43.65%
PT2	65037488	55445945	85.25%	52738564	81.09%	27563636	42.38%	27476279	43.65%
PT3	65316422	56957569	87.2%	54089388	82.81%	28293609	43.32%	28228884	43.22%
PT4	75506064	64269152	85.12%	61021292	80.82%	31904145	42.25%	31888064	42.23%
PT5	68292034	53465050	78.29%	50826310	74.42%	26552333	42.25%	26493568	38.79%
PX1	50908394	43024726	84.51%	40845785	80.23%	21424253	42.08%	21316946	41.87%
PX2	67418530	58984363	87.49%	55702668	82.62%	29322172	43.49%	29258327	43.40%
PX3	59116686	51374316	86.90%	48593438	82.20%	25530131	43.19%	25474898	43.09%
PX4	50070956	42965914	85.81%	40786889	81.46%	21351653	42.64%	21342061	42.62%
PX5	58923046	51046402	86.63%	48489963	82.29%	25375162	43.06%	25318363	42.97%

## Data Availability

The data used to support the findings of this study are available from the corresponding author upon request.

## Supplementary Materials

Supplementary Materials Table S1: primer sequences for the genes selected for qRT-PCR. Table S2: DEGs identified under certain filter criteria. Table S3: GO enrichment analysis of DEGs. Table S4: KEGG pathway analysis of DEGs.

## References

[1] Bae Y. S., Lee J. C., Jung S. (2014). Differentiation of deboned fresh chicken thigh meat from the frozen-thawed one processed with different deboning conditions. *Korean Journal for Food Science of Animal Resources*.

[2] Skunca D., Tomasevic I., Zdolec N., Kolaj R., Aleksiev G., Djekic I. (2017). Consumer-perceived quality characteristics of chicken meat and chicken meat products in Southeast Europe. *British Food Journal*.

[3] Franke C., Holl L., Langowski H. C., Petermeier H., Vogel R. F. (2017). Sensory evaluation of chicken breast packed in two different modified atmospheres. *Food Packaging and Shelf Life*.

[4] Hejdysz M., Kaczmarek S. A., Rogiewicz A., Rutkowski A. (2019). Influence of graded levels of meals from three lupin species on growth performance and nutrient digestibility in broiler chickens. *British Poultry Science*.

[5] Mir N. A., Rafiq A., Kumar F., Singh V., Shukla V. (2017). Determinants of broiler chicken meat quality and factors affecting them: a review. *Journal of Food Science and Technology*.

[6] Zhang M., Li F., Ma X.-f. (2019). Identification of differentially expressed genes and pathways between intramuscular and abdominal fat-derived preadipocyte differentiation of chickens in vitro. *BMC Genomics*.

[7] Felício A. M., Gaya L. G., Ferraz J. B. S. (2013). Heritability and genetic correlation estimates for performance, meat quality and quantitative skeletal muscle fiber traits in broiler. *Livestock Science*.

[8] Lee S. H., Joo S. T., Ryu Y. C. (2010). Skeletal muscle fiber type and myofibrillar proteins in relation to meat quality. *Meat Science*.

[9] Han S., Cui C., Wang Y. (2019). FHL3 negatively regulates the differentiation of skeletal muscle satellite cells in chicken. *3 Biotech*.

[10] Stein J. M., Padykula H. A. (1962). Histochemical classification of individual skeletal muscle fibers of the rat. *American Journal of Anatomy*.

[11] Bassel-Duby R., Olson E. N. (2006). Signaling pathways in skeletal muscle remodeling. *Annual Review of Biochemistry*.

[12] Petracci M., Sirri F., Mazzoni M., Meluzzi A. (2013). Comparison of breast muscle traits and meat quality characteristics in 2 commercial chicken hybrids. *Poultry Science*.

[13] Raj S., Skiba G., Weremko D. (2010). The relationship between the chemical composition of the carcass and the fatty acid composition of intramuscular fat and backfat of several pig breeds slaughtered at different weights. *Meat Science*.

[14] Lu Z. Q., Ren Y., Zhou X. H. (2017). Maternal dietary linoleic acid supplementation promotes muscle fibre type transformation in suckling piglets. *Journal of Animal Physiology and Animal Nutrition*.

[15] Zhu H., Yang H., Zhao W., Su Y., Tian Y. (2019). Associations of the expression levels of genes involved inCFL2bandMyHCisoform type changes in longissimus dorsi muscle of HeBao and Large White pigs (Sus scrofa) during postnatal growth. *Canadian Journal of Animal Science*.

[16] Zhang Y., Yan H., Zhou P., Zhang Z., Liu J., Zhang H. (2019). MicroRNA-152 promotes slow-twitch myofiber formation via targeting uncoupling protein-3 gene. *Animals*.

[17] Shu J. T., Xu W. J., Zhang M. (2014). Transcriptional co-activator PGC-1*α* gene is associated with chicken skeletal muscle fiber types. *Genetics and Molecular Research*.

[18] Ma M., Cai B., Jiang L. (2018). lncRNA-Six1 is a target of miR-1611 that functions as a ceRNA to regulate Six1 protein expression and fiber type switching in chicken myogenesis. *Cells*.

[19] Ghosh M., Sharma N., Singh A. K., Gera M., Pulicherla K. K., Jeong D. K. (2018). Transformation of animal genomics by next-generation sequencing technologies: a decade of challenges and their impact on genetic architecture. *Critical Reviews in Biotechnology*.

[20] Zhang C., Wang G., Wang J. (2013). Characterization and comparative analyses of muscle transcriptomes in Dorper and small-tailed Han sheep using RNA-Seq technique. *Plos One*.

[21] Fonseca L. F. S., Dos Santos Silva D. B., Gimenez D. F. J. (2020). Gene expression profiling and identification of hub genes in Nellore cattle with different marbling score levels. *Genomics*.

[22] Song S.-Q., Ma W.-w., Zeng S.-X. (2019). Transcriptome analysis of differential gene expression in the longissimus dorsi muscle from Debao and landrace pigs based on RNA-sequencing. *Bioscience Reports*.

[23] Piórkowska K., Żukowski K., Nowak J., Połtowicz K., Ropka-Molik K., Gurgul A. (2016). Genome-wide RNA-Seq analysis of breast muscles of two broiler chicken groups differing in shear force. *Animal Genetics*.

[24] Dong H., Wang J. J., Ying T. Y., Ting L. I., Wang H. L. (2005). Extraction of total RNA and protein by the method of TRIzol. *Letters in Biotechnology*.

[25] Kim D., Langmead B., Salzberg S. L. (2015). HISAT: a fast spliced aligner with low memory requirements. *Nature Methods*.

[26] Pertea M., Pertea G. M., Antonescu C. M., Chang T.-C., Mendell J. T., Salzberg S. L. (2015). StringTie enables improved reconstruction of a transcriptome from RNA-seq reads. *Nature Biotechnology*.

[27] Florea L., Song L., Salzberg S. L. (2013). Thousands of exon skipping events differentiate among splicing patterns in sixteen human tissues. *F1000res*.

[28] Dona M. S. I., Prendergast L. A., Mathivanan S., Keerthikumar S., Salim A. (2017). Powerful differential expression analysis incorporating network topology for next-generation sequencing data. *Bioinformatics*.

[29] Anders S., Huber W. (2010). Differential expression analysis for sequence count data. *Genome Biology*.

[30] Young M. D., Wakefield M. J., Smyth G. K., Oshlack A. (2010). Gene ontology analysis for RNA-seq: accounting for selection bias. *Genome biology*.

[31] Mao X., Cai T., Olyarchuk J. G., Wei L. (2005). Automated genome annotation and pathway identification using the KEGG Orthology (KO) as a controlled vocabulary. *Bioinformatics*.

[32] Luo W., Wu H., Ye Y. (2014). The transient expression of miR-203 and its inhibiting effects on skeletal muscle cell proliferation and differentiation. *Cell Death & Disease*.

[33] Hu H. M., Wang J. Y., Zhu R. S., Guo J. F., Wu Y. (2008). Effect of myosin heavy chain composition of muscles on meat quality in Laiwu pigs and Duroc. *Science in China. Series C, Life Sciences*.

[34] Sanchez H., Chapot R., Banzet S. (2006). Quantification by real-time PCR of developmental and adult myosin mRNA in rat muscles. *Biochemical and biophysical research communications*.

[35] Ahhmed A. M., Birisik C., Kaneko G. (2015). Differences in gelling properties induced by transglutaminase in chicken muscles are explained by determining myosin heavy chain mRNA ratios using RT-PCR technique. *Fleischwirtschaft*.

[36] Li Y., Yuan L., Yang X. (2007). Effect of early feed restriction on myofibre types and expression of growth-related genes in the gastrocnemius muscle of crossbred broiler chickens. *The British journal of nutrition*.

[37] Amann R., Wyder S., Slavotinek A. M., Trueb B. (2014). The FgfrL1 receptor is required for development of slow muscle fibers. *Developmental Biology*.

[38] Machida S., Noda S., Takao A., Nakazawa M., Matsuoka R. (2002). Expression of slow skeletal myosin heavy chain 2 gene in Purkinje fiber cells in chick heart. *Biology of the cell*.

[39] Fang-Hua L. I., Hou L. L., Yue-Hui M. A., Pang Q. H., Guan W. J. (2010). Isolation, culture, identification and muscle differentiation of skeletal muscle satellite cells in Beijing fatty chicken. *Scientia Agricultura Sinica*.

[40] Jurie C., Picard B., Heyman Y. (2009). Comparison of cloned and non-cloned Holstein heifers in muscle contractile and metabolic characteristics. *Animal*.

[41] Dai F., Feng D., Cao Q. (2010). Developmental differences in carcass, meat quality and muscle fibre characteristics between the Landrace and a Chinese native pig. *South African Journal of Animal Science*.

[42] Taglietti V., Maroli G., Cermenati S. (2016). Nfix induces a switch in Sox6 transcriptional activity to regulate MyHC-I expression in fetal muscle. *Cell Reports*.

[43] Yu L.-H., Lee E.-S., Chen H.-S. (2011). Comparison of physicochemical characteristics of hot-boned chicken breast and leg muscles during storage at 20°C. *Korean Journal for Food Science of Animal Resources*.

[44] Loughna P. T., Mason P., Bayol S., Brownson C. (2000). The LIM-domain protein FHL1 (SLIM 1) exhibits functional regulation in skeletal muscle. *Molecular Cell Biology Research Communications*.

[45] Cowling B. S., McGrath M. J., Nguyen M.-A. (2008). Identification of FHL1 as a regulator of skeletal muscle mass: implications for human myopathy. *Journal of Cell Biology*.

[46] Hendricks H. B., Lafferty D. T., Aberle E. D., Judge M. D., Forrest J. C. (1971). Relation of porcine muscle fiber type and size to postmortem shortening. *Journal of Animal Science*.

[47] Capalbo G., Müller-Kuller T., Ottmann O. G., Hoelzer D., Scheuring U. J. (2012). HIV-1 infection suppresses expression of host cell cycle-associated GenePDS5A. *Intervirology*.

[48] Ropka-Molik K., Bereta A., Żukowski K., Piórkowska K., Gurgul A., Żak G. (2017). Transcriptomic gene profiling of porcine muscle tissue depending on histological properties,. *Animal Science Journal*.

[49] Tee J.-M., van Rooijen C., Boonen R., Zivkovic D. (2009). Regulation of slow and fast muscle myofibrillogenesis by Wnt/*β*-catenin and myostatin signaling. *Plos One*.

[50] Angonin D., Van Raay T. J. (2013). Nkd1 functions as a passive antagonist of Wnt signaling. *PLoS One*.

[51] Conti A., Gorza L., Sorrentino V. (1996). Differential distribution of ryanodine receptor type 3 (RyR3) gene product in mammalian skeletal muscles. *Biochemical Journal*.

[52] Perez C. F., López J. R., Allen P. D. (2005). Expression levels of RyR1 and RyR3 control resting free Ca2+ in skeletal muscle. *American Journal of Physiology. Cell Physiology*.

[53] Danieli-Betto D., Betto R., Midrio M. (1990). Calcium sensitivity and myofibrillar protein isoforms of rat skinned skeletal muscle fibres. *Pflügers Archiv European Journal of Physiology*.

[54] Nicol C. J. M., Johnston I. A. (1981). Energy metabolism of fast- and slow-twitch skeletal muscle in the rat: thyroid hormone induced changes. *Journal of Comparative Physiology*.

[55] Choi Y. M., Ryu Y. C., Kim B. C. (2007). Influence of myosin heavy- and light chain isoforms on early postmortem glycolytic rate and pork quality. *Meat Science*.

[56] Rizk N. M., Meier D. A., Pastorek D. J., Krakower G. R., Kissebah A. H. (1998). Glucose utilization in muscle fiber types: use of the partial pancreatectomized rat model to distinguish effects of glucose and insulin on insulin resistance. *Molecular Genetics and Metabolism*.

[57] Liu J., Fu R., Liu R. (2016). Protein profiles for muscle development and intramuscular fat accumulation at different post-hatching ages in chickens. *Plos One*.

[58] Xiong Y., Lei Q.-Y., Zhao S., Guan K.-L. (2012). Regulation of glycolysis and gluconeogenesis by acetylation of PKM and PEPCK. *Cold Spring Harbor Symposia on Quantitative Biology*.

[59] Zhan C., Yan L., Wang L. (2015). Isoform switch of pyruvate kinase M1 indeed occurs but not to pyruvate kinase M2 in human tumorigenesis. *Plos One*.

[60] Schwägele F., Haschke C., Krauss G., Honikel K. O. (1996). Comparative studies of pyruvate kinase from PSE and normal pig muscles. *Zeitschrift für Lebensmittel-Untersuchung und -Forschung*.

[61] Van Hall G. (2000). Lactate as a fuel for mitochondrial respiration. *Acta Physiologica Scandinavica*.

[62] Qiu H., Xu X., Fan B., Rothschild M. F., Martin Y., Liu B. (2010). Investigation of LDHA and COPB1 as candidate genes for muscle development in the MYOD1 region of pig chromosome 2. *Molecular Biology Reports*.

[63] Liu J., Lei Q., Li F. (2020). Dynamic transcriptomic analysis of breast muscle development from the embryonic to post-hatching periods in chickens. *Frontiers in Genetics*.

[64] Taha C., Klip A. (1999). The insulin signaling pathway.

[65] Dimitriadis G., Mitrou P., Lambadiari V., Maratou E., Raptis S. A. (2011). Insulin effects in muscle and adipose tissue. *Diabetes Res Clin Pract*.

[66] Choe J. H., Choi Y. M., Lee S. H. (2008). The relation between glycogen, lactate content and muscle fiber type composition, and their influence on postmortem glycolytic rate and pork quality. *Meat Science*.

[67] Huang X., Vaag A., Hansson M., Weng J., Laurila E., Groop L. (2000). Impaired insulin-stimulated expression of the glycogen synthase gene in skeletal muscle of type 2 diabetic patients is acquired rather than inherited. *Journal of Clinical Endocrinology & Metabolism*.

[68] Nogales-Gadea G., Mormeneo E., García-Consuegra I. (2010). Expression of glycogen phosphorylase isoforms in cultured muscle from patients with McArdle's disease carrying the p.R771PfsX33 PYGM mutation. *PloS one*.

[69] Xu N., Geller D. H., Jones M. R., Funari V. A., Azziz R., Goodarzi M. O. (2015). Comprehensive assessment of expression of insulin signaling pathway components in subcutaneous adipose tissue of women with and without polycystic ovary syndrome. *Journal of Clinical & Translational Endocrinology*.

[70] Francisco C. L., Jorge A. M., Dal-Pai-Silva M., Carani F. R., Cabeço L. C., Silva S. R. (2011). Muscle fiber type characterization and myosin heavy chain (MyHC) isoform expression in Mediterranean buffaloes. *Meat Science*.

[71] Bandman E., Rosser B. W. C. (2000). Evolutionary significance of myosin heavy chain heterogeneity in birds. *Microscopy Research and Technique*.

[72] Bai C., Hou L., Li F., He X., Zhang M., Guan W. (2012). Isolation and biological characteristics of Beijing fatty chicken skeletal muscle satellite cells. *Cell Communication and Adhesion*.

[73] Brown D. M., Parr T., Brameld J. M. (2012). Myosin heavy chain mRNA isoforms are expressed in two distinct cohorts during C2C12 myogenesis. *Journal of Muscle Research and Cell Motility*.

